# Malaria Mortality in Brazil: Age–Period–Cohort Effects, Sociodemographic Factors, and Sustainable Development Indicators

**DOI:** 10.3390/tropicalmed10020041

**Published:** 2025-01-31

**Authors:** Mariusa Fernandes de Farias, Eric Renato Lima Figueiredo, Raimundo Nelson Souza da Silva, Deizyane dos Reis Galhardo, Cleide Laranjeira da Silva, Evelyn Myelle Farias Moreira, Yury Souza de Azevedo, Emilly Cassia Soares Furtado, Janielly Reis Castelhano, João Simão de Melo-Neto, Fabiana de Campos Gomes

**Affiliations:** 1 The Institute of Health Sciences (ICS), Federal University of Pará (UFPA), Belém 66075-110, PA, Brazil; mestradosaudecoletiva2022@gmail.com (M.F.d.F.); eric.renatoo@gmail.com (E.R.L.F.); deizyanegalhardo@gmail.com (D.d.R.G.); cleidedasilva.20@gmail.com (C.L.d.S.); evelynmoreirappgcmh.ufpa@gmail.com (E.M.F.M.); yurysouza0701@gmail.com (Y.S.d.A.); emilly.furtado@ics.ufpa.br (E.C.S.F.); janiecastelhano@gmail.com (J.R.C.); facamposgomes@gmail.com (F.d.C.G.); 2 The Institute of Animal Health and Production (ISPA), Federal Rural University of Amazonia (UFRA), Belém 66077-830, PA, Brazil; ispa@ufra.edu.br; 3 Ceres Medical School (FACERES), Campus FACERES, São José do Rio Preto 15090-305, SP, Brazil

**Keywords:** inequity, malaria, mortality, Brazil, public health

## Abstract

Introduction: Human malaria is a zoonosis considered a serious global public health problem caused by five species of protozoa of the genus *Plasmodium* spp., which are transmitted by mosquito vectors belonging to the genus *Anopheles* spp. Objective: To verify whether there is a relationship between the age-standardized malaria mortality rate in Brazil and age–period–cohort effect variables, sociodemographic differences, and indicators of sustainable development. Methods: Data on malaria mortality in Brazil from 2000 to 2022 were analyzed using sociodemographic factors such as ethnicity, region of residence, and sustainable development indicators. Results: Statistical data demonstrated that from 2000 to 2022, there was a reduction in malaria mortality; the 0–4 years age group was more susceptible to death, and the infection affected more men, Indigenous people, and residents of the North Region. Environmental factors such as CO_2_ emissions and sanitation predict mortality in specific regions. The North and Northeast Regions had higher mortality rates. In the North, low CO_2_ emissions, deforestation, weak urban sanitation, a lower GDP, and a higher Gini index were related to high mortality; the latter was also a factor in high rates of deforestation and solid waste collection in the Central West and Northeast Regions. The number of consultations and professionals was a predictive factor for high rates in the three regions mentioned. The Southeast Region had the lowest mortality rate and lowest health expenditure, while the Northeast and Midwest Regions had the highest expenditure in this sector. Conclusions: This study can contribute to the direction of public policy due to the specificities of each region in Brazil.

## 1. Introduction

Malaria is an infectious disease caused by *Plasmodium* spp. and is transmitted by infected *Anopheles* spp. mosquitoes. Although it remains one of the main epidemiological concerns in Brazil, it is preventable and curable [1]. Its complex chain of transmission involves dynamic interactions between humans, mosquitoes, parasites, the environment, health systems, and public policies [2,3]. Despite advances in treatment, malaria treatment continues to challenge health systems due to the high mortality rate in endemic areas, with a varied epidemiological pattern in these regions [3].

Data from the World Health Organization (WHO) indicate that approximately 247 million cases and 627,000 deaths have occurred globally as a result of malaria in recent years [4]. In Brazil, between 2019 and 2020, there was a 10.5% reduction in cases. Following the national malaria eradication campaigns in the late twentieth century, over 99% of malaria cases are now confined to the Amazon region [5].

Despite advances in antimalarial drugs, treatment remains a major challenge for health systems, directly impacting endemic areas and the diversity of epidemiological patterns in these regions. Human activities that alter the environment have considerably increased the risk of malaria transmission, affecting the disease’s vector populations [6]. Even with such an impact, no studies in the literature have assessed the relationship between mortality and sustainable development indicators.

Thus, this study aimed to verify whether the age-standardized mortality rate from malaria in Brazil is related to the age–period–cohort effect, sociodemographic differences, and sustainable development indicators. Our initial hypothesis was that individuals with malaria die more frequently when they fulfil the following criteria: young adults, due to greater exposure to breeding sites; those born after the 1970s and more frequently in recent years, following an increase in deforestation; mainly those who are black and brown; those living in northern Brazil; and those with worse sustainable development indicators.

## 2. Methods

### 2.1. Ethical Aspects

This study used secondary data on malaria mortality available in public-domain databases. It did not require the approval of the Research Ethics Committee, as per Resolution 738 of 1 February 2024 of the National Health Council, which provides for the use of databases for scientific research involving human beings [7].

### 2.2. Type of Study

This was an ecological observational study with a descriptive and inferential analytical approach [8].

### 2.3. Population, Area, and Study Period

**Figure 1:**
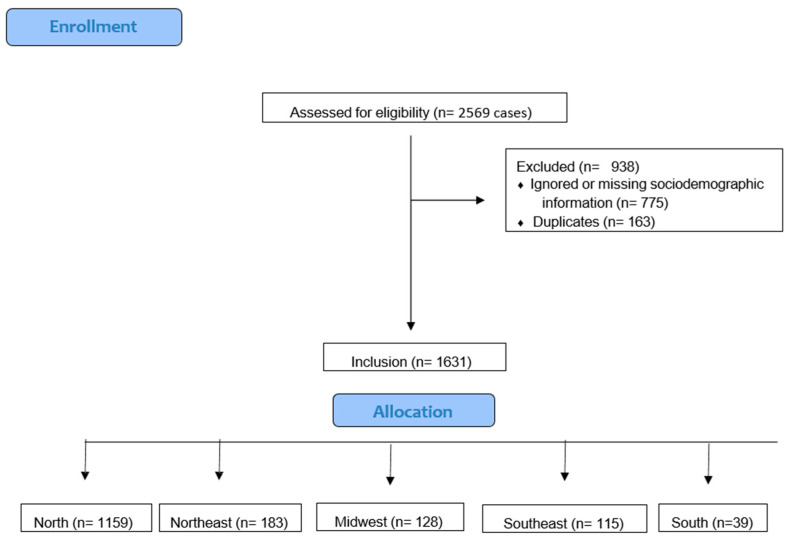
Data eligibility flow diagram.

This population-based study consisted of individuals of all ages who were registered as having died from malaria in the Ministry of Health’s Mortality Information System (SIM). Data on deaths with the underlying cause of death attributed to malaria, as defined by ICD-10 codes B50 to B54, were included. A total of 2569 cases were initially included. Applying the inclusion and exclusion criteria from Figure 1, cases with missing sociodemographic information and duplicates were excluded, resulting in a final sample of 1631 observations aggregated by macro-region of residence from 1996 to 2022.

**Figure 2:**
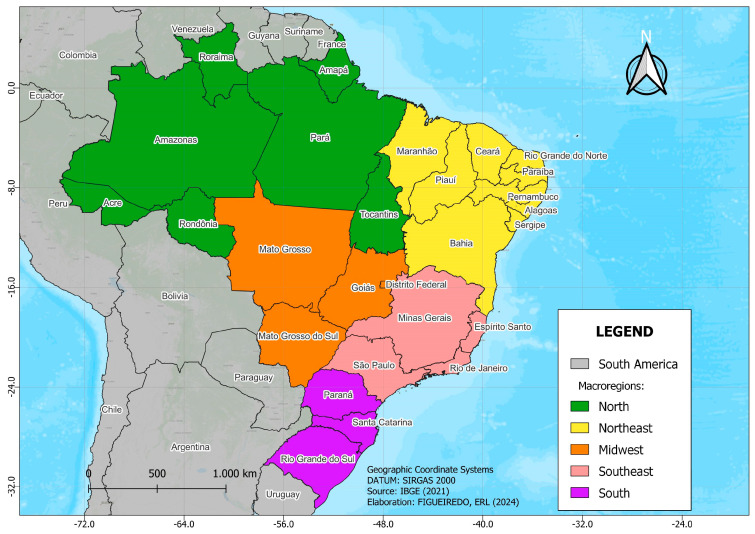
States and geographic regions of Brazil.

The study area (Figure 2) covered the Brazilian territory, considering the political–administrative division into federal units and five macroregions: North, Northeast, Midwest, Southeast, and South. The analysis was stratified to allow comparisons between the different regions. Depending on the availability and completion of data, the analysis period extended from 6 April 1997 to 27 October 2022. This time interval was selected to provide a comprehensive view of the evolution of malaria mortality over more than two decades.

### 2.4. Database

The study data were obtained from two databases: (1) the Department of Information and Informatics of the Unified Health System (DATASUS) through the Tabulation Tool (TABNET) [9] and (2) the Brazilian Institute of Geography and Statistics (IBGE) through the IBGE System of Automatic Recovery (SIDRA) [10].

Through TABNET, we were able to access the Mortality Information System (SIM) and the Basic Indicators and Data (IDB) databases. On the other hand, the SIDRA gave us access to sustainable development indicators (IDSs). Table 1 provides details about the variables collected, descriptions, and specific analysis periods for each database. The study variables are described according to their characteristics, data sources, and collection periods.

### 2.5. Study Variables

First, the absolute number of deaths was collected from the SIM, and the total resident population by region was obtained from the IBGE to analyze the effects of age, event period, and birth cohort.

Next, the study’s dependent variable, the age-standardized mortality rate, was developed using data on malaria deaths, concerning the independent variables of race/color, region, and the world’s average population [11]. The IBGE classification indicates that race/ethnicity is self-reported. In addition, distinctions between groups in terms of ethnic, linguistic, cultural, or historical characteristics were not clearly defined; therefore, any attempt to categorize people as Caucasian or Hispanic in Brazil may be inaccurate. The demographic variables considered were geographic regions (North, Northeast, Southeast, South, and Midwest).

Age standardization allows direct comparisons between different populations in regions by adjusting for variations in age distribution, thus eliminating the influence of age structure on mortality [12].

The sustainable development indicators were analyzed based on three dimensions, environmental, socioeconomic, and institutional [10], to verify their relationships with the age-standardized mortality rate according to each region of Brazil. In the environmental dimension, the following variables were analyzed: urbanization, anthropogenic carbon dioxide emissions, number of hotspots, annual gross deforestation in the legal Amazon/km^2^, percentage of garbage collection in urban areas, percentage of garbage collection in rural areas, and access to public sewerage. In the socioeconomic dimension, gross domestic product (GDP) and the Gini index were analyzed. In the institutional dimension, the following were studied: the number of health professionals per inhabitant, expenditures on public health measures and services, and consultations per inhabitant per region.

These indicators were considered independent variables in this study. The analysis of these indicators will allow us to understand the dynamics that determine and condition [13] the deaths in Brazil caused by this neglected tropical disease.

### 2.6. Statistical Analysis

To identify the influence of age, period of death, and birth cohort on mortality from malaria, the APC Web tool Biostatistics Branch, National Cancer Institute, Bethesda, MD, USA [14] was used. As described by Nascimento et al. [15], the following variables were analyzed: net drift; all age or period or cohort deviations; all period or cohort rate ratios (RR); and all local drift. The APC model incorporates parameters such as trends and deviations that depict the mathematical interactions between cancer incidence, age, year of diagnosis, and year of birth. It allows for the estimation of how the effects of age, period, and birth cohort influence age-adjusted malaria mortality. Data on deaths and the vulnerable population were organized into regular 5-year intervals to constrain the estimated parameters. Eighteen age groups were considered (ranging from 0–4 years to 75–89 years), four periods (from 1996–2004 to 2015–2019), and 21 birth cohorts, each spanning 5 years (from 1915 to 2015). Variations were considered statistically significant with (*p* < 0.05) through the Wald test (hypothesis test). For all variables analyzed, the following functions were estimated: net drifts (overall annual percentage change according to the calendar and birth cohort), local drifts (annual percentage changes for each age group, also considering the calendar and birth cohort), all age deviations (adjusted longitudinal and cross-sectional curves present a log-linear relationship), all period deviations (adjusted temporal trends and period rates have a log-linear format), all cohort deviations (cohort rates are log-linear and all local drifts equal net drifts), and all period (or cohort) rate ratios (RR; age incidence pattern in each period (or cohort)).

During the intergroup comparison, after applying the Shapiro-Wilk test to verify the normality of the data, the Kruskal–Wallis test (nonparametric) was used to determine whether there were statistically significant differences between the medians of the age-standardized mortality rates of the race and geographical region subgroups.

To develop the models corresponding to each sociodemographic group, the necessary assumptions for multiple linear regression were observed. First, the significance of each model was verified by analyzing the generated F-statistic and performing a hypothesis test. The null hypothesis that all regression coefficients (β) are statistically equal to zero (indicating that there is no regression model) was rejected, indicating the existence of a significant regression model (*p* < 0.05). Next, Durbin–Watson analysis was performed to check for autocorrelation of the residuals of the model. The results were within the acceptable range of 1.5 to 2.5, indicating that the residuals are independent. Collinearity was assessed by considering tolerance (values greater than 0.2) and VIF (values less than 10) to ensure that the independent variables were not highly correlated. The analysis of the standardized residuals confirmed the absence of outliers, as the values were between −3 and +3. We then examined the interdependence between the residuals and Cook’s distance for each observation. The range considered adequate for the interdependence of the residuals varied from 1.5 to 2.5, while Cook’s distance should be less than 1.

## 3. Results

During the period from 1996 to 2022, 2569 cases of mortality caused by malaria were registered in the mortality surveillance system in Brazil. Considering the quality of the data by aggregating the 1631 cases with the completion of all socio-demographic variables of interest for the research, we highlight here the percentage of completeness for each variable: sex/gender—100%; race/color—79%; marital status—84%; educational level—67%; place of occurrence—100%.

**Figure 3:**
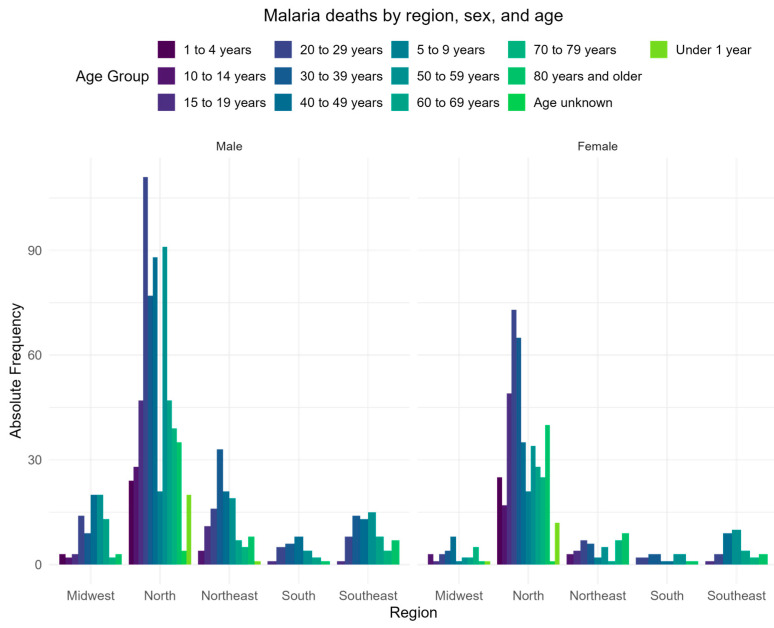
Malaria deaths categorized by sex and age group for each region.

Table 2 contains the aggregated information about the cohort, and Figure 3 presents the number of deaths categorized by sex and age group for each region. This visualization provides a detailed breakdown of mortality distribution across different demographic segments within the studied areas.

### 3.1. Age–Period–Cohort Effect

During the period 1996–2022, the annual percentage change in the expected age-adjusted rates (net drift) was −9.37% (95% CI: −10.35, −8.39) per year (χ^2^ = 316.87, *p* < 0.001).

**Figure 4:**
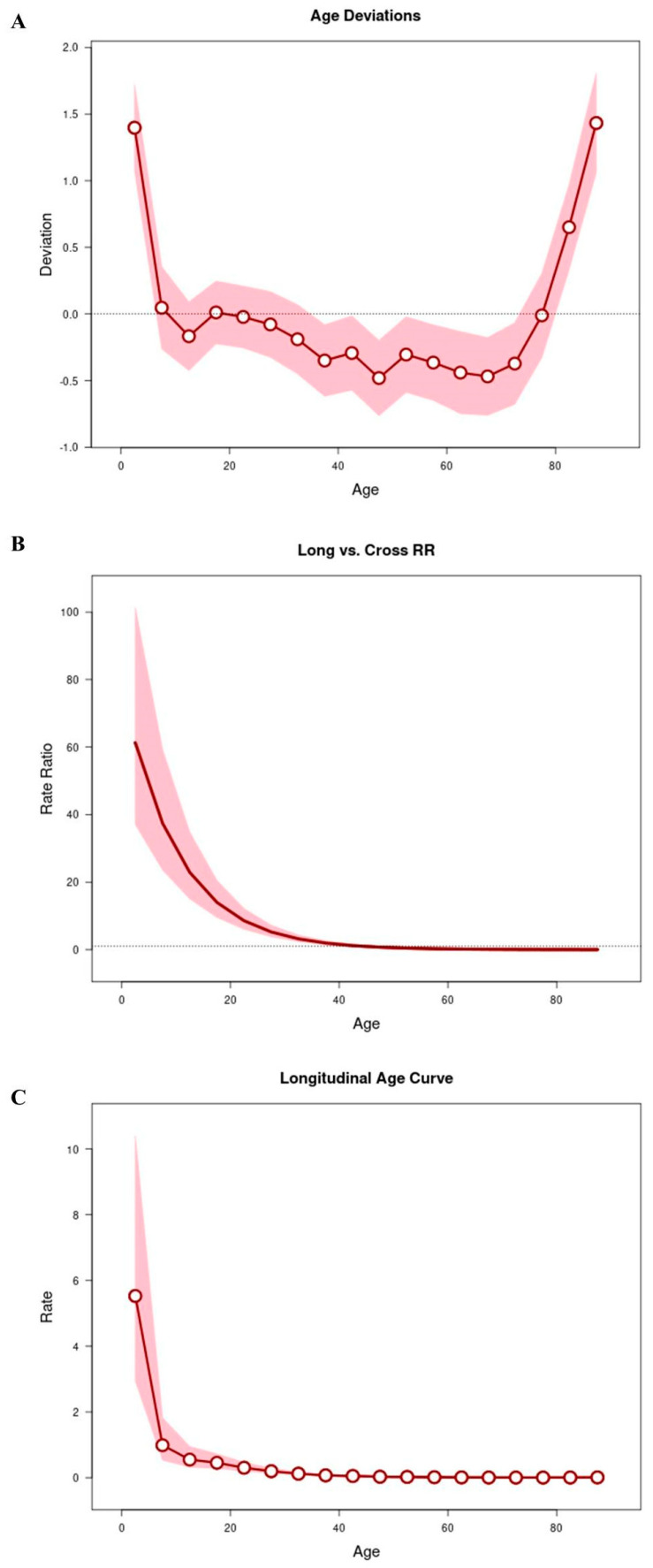
APC analysis to assess whether age is a determining factor in the number of deaths. All age deviations are shown in (**A**). The age curves are log-linear (**B**). Longitudinal age curves (**C**).

Figure 4 shows the results obtained from the APC analysis to assess whether age is a determining factor in mortality. All age deviations (χ^2^ = 151.65, *p* < 0.001) (Figure 4A) showed that the adjusted longitudinal and transverse age curves were log-linear (Figure 4B). However, we observed that only the longitudinal age curves had a rate greater than 1 in the 0 to 4 years age group [RR: 5.53; 95% CI: 2.94, 10.40], as shown in Figure 4C.

**Figure 5:**
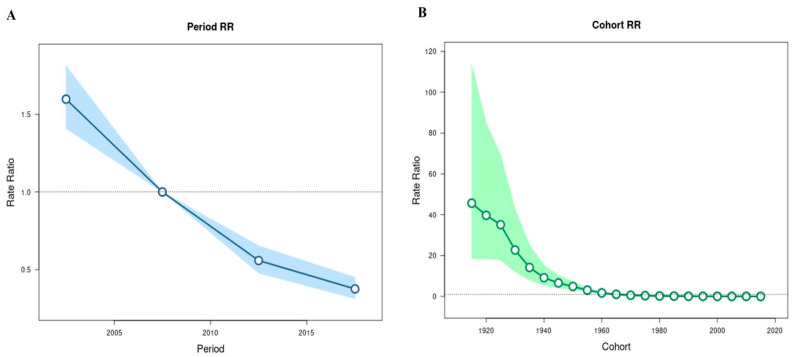
APC analysis was performed to assess whether the period (**A**) or cohort (**B**) are determining factors for the rate of death.

All period RRs were significant (χ^2^ = 366.41, *p* < 0.001) (Figure 5A), showing that the period between 2000 and 2004 (Rate: 1.60; 95% CI: 1.41, 1.81) had a greater RR. All cohort rates (χ^2^ = 369.59, *p* < 0.0001) (Figure 5B) were significant, showing that individuals born before 1960 (rate > 1.70; 95% CI > 1.27, 2.30) had higher mortality.

### 3.2. Regional and Racial Differences

**Figure 6:**
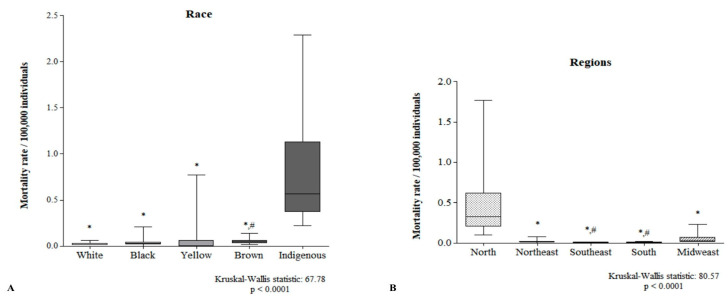
Racial (**A**) and regional (**B**) differences in the rate of death. In (**A**), * *p* < 0.01 compared to Iindigenous people and # *p* < 0.01 compared to yellow people. In (**B**), * *p* < 0.01 compared to the North Region and # *p* < 0.001 compared to the Midwest Region.

There was a higher mortality rate among Indigenous people than among people of other races. In addition, brown people died more than yellow people (Figure 6A). The mortality rate was greater in the North region than in the other regions (Figure 6B).

### 3.3. Sustainable Development Indicators

#### 3.3.1. Environmental Dimension

The lower degree of urbanization in the North, Northeast, and South Regions was related to a higher mortality rate (Table 3). There was an inversely proportional relationship between anthropogenic carbon dioxide emissions and the mortality rate in the North, Northeast, and South Regions (Table 3). Higher levels of deforestation in the North and Northeast Regions were associated with higher mortality (Table 3). The lower the garbage collection rate in urban areas was, the greater the mortality rate in the North and Northeast Regions (Table 3). There was a relationship between lower garbage collection rates in rural areas and higher mortality in the Northeast Region (Table 3). Access to sanitation in urban areas was inversely correlated with this rate in the North, Northeast, and South Regions (Table 3).

The prediction models showed that (1) lower anthropogenic carbon dioxide emissions are proportional to a higher mortality rate in the North Region and (2) greater access to sanitation in urban areas is related to lower mortality rates in the South Region (Table 4).

#### 3.3.2. Socioeconomic Dimension

Areas (North, Northeast, and Midwest) with lower GDP per capita were related to higher mortality (Table 3). Higher Gini index values per capita were related to higher mortality in the North, Northeast, and Midwest Regions (Table 3).

In addition, the linear regression model showed that in the North, the lowest GDP per capita was a predictor of higher mortality (Table 3). The highest Gini index per capita was a predictor of higher mortality in the North and Midwest Regions (Table 4).

#### 3.3.3. Institutional Dimension

A lower number of health professionals per inhabitant and consultations per inhabitant per region was related to a higher mortality rate in the North, Northeast, and Midwest Regions (Table 3). Spending on public health actions and services was lower in the Northeast and Midwest Regions, resulting in higher mortality, while in the Southeast Region, low spending was accompanied by lower rates of death (Table 4).

## 4. Discussion

Malaria still represents a significant public health challenge in the country, especially in the Amazon region [16]. According to Meireles et al. [17], men, particularly those who are Indigenous, have an unfavorable outcome, as indicated by their behavior and way of life based on extractives, which leaves them more exposed to vectors.

Probably as a result of the early diagnosis and treatment of cases and the decrease in *P. falciparum* transmission, the number of hospitalizations due to malaria decreased (from 53,450 in 1994 to 18,037 in 2000 and 4442 in 2009), as did the number of recorded deaths attributed to the disease (from 897 in 1984 to 58 in 2009) and the case fatality rate (from 0.038% in 2000 to 0.013% in 2009) in the Amazon [18].

According to the WHO, some population groups, including infants and children under 5 years of age, have a greater risk of severe malaria infection [19,20]. This information is related to the results presented in this study, which showed a higher mortality rate from malaria in the age group from 0 to 4 years in the period from 2000 to 2004 in Brazil. This may be related to the low functional immunity and developing neurological system in this age group, in addition to contributing more to the parasite reservoir [20], increasing susceptibility to severe infections and complications of malaria.

Reports on malaria in Brazil indicate that after the implementation of plans to intensify actions to control the disease in the 2000s, there was an initial reduction in cases [21]. However, subsequently, a significant increase in the number of cases was observed, which continued to increase up to 2005 [22]. This scenario of alternation between a decline and resurgence of malaria may help to explain the peak mortality observed in the age group of 0 to 4 years in the period from 2000 to 2004 in the country. These data reinforce the need to prioritize ongoing prevention and treatment strategies to protect these vulnerable groups against malaria.

Regarding racial differences, Indigenous people had the highest mortality rate compared to people of other races. In addition to environmental and socioeconomic issues, which affect Indigenous and nonindigenous people [23], this high rate may be related to the access and health coverage of this population, which is hindered by geographical isolation [24]. In this sense, studies have shown that Indigenous populations have factors that affect their health regarding malaria, such as traditional dwellings that hinder vector control methods [5], the severity of the disease due to contact with *P. falciparum* [14], and human mobility, which is marked by the circulation of parasites with introductions and reintroductions in different territories [21].

In Brazil, regional disparities are known to contribute to different patterns of distribution of malaria mortality; considering this, the results of this study revealed a higher mortality rate due to malaria in the North Region of Brazil than in other regions. These results are similar to those of other studies [25] that identified a reduction in malaria deaths; however, the highest number of cases occurred in the North Region. This may be related to the geographical characteristics of the legal Amazon, which covers all the states in the North Region [26] and provides favorable environmental conditions for the transmission of the disease, such as the presence of *Anopheles* spp. mosquitoes, and the lack of effective control over the spread of the disease, even after implementing control and prevention programs.

Although the Amazon region is the main malaria-endemic area in Brazil, the disease is also present in other regions of the country and is reported as imported malaria [27]. In these locations, malaria is predominantly caused by *Plasmodium vivax*, with a significant proportion of cases of double infection with *P. falciparum*, mainly in the Midwest Region [16]. Although the total number of malaria deaths in the extra-Amazon region is lower than in the legal Amazon region, the relative lethality was 123 times higher than in the Amazon region in 2019 [28].

These characteristics of imported malaria are worrisome, as only 19% of malaria cases occurring in nonendemic regions are diagnosed and treated early, in contrast to 60% of incident cases in the Amazon [16]. This may contribute to the greater severity of cases and, consequently, to the higher mortality observed in the Midwest region than in other extra-Amazonian regions. We suggest here the importance of further studies on parasite species and malaria mortality. In addition, the information system and mortality surveillance service should include information on parasite species. In this sense, the lack of this information is characterized as a limitation of this research and an improvement of the granularity of the data available in the Brazilian mortality information system.

Studies on the effects of urbanization on the distribution of malaria vectors in areas with human intervention and forest regions in the North revealed that the largest mosquito population was found in areas with clean water and vegetation covered by trees [29]. In this context, another study comparing the distribution of malaria vectors in areas with human intervention and forest regions in the North Region revealed that the largest mosquito population was found in areas with clean water and vegetation covered by trees [30].

Our study revealed an inverse relationship between CO_2_ emissions and mortality rates in the North, Northeast, and South Regions of Brazil. This same trend is observed in countries in rural Africa, a region with high malaria mortality but that contributes little to global carbon emissions [31]. In addition, our predictive model indicated that lower CO_2_ emissions are associated with higher mortality rates, especially in the North Region. This suggests that this region, which has one of the lowest industrial development levels [32] and, consequently, lower CO_2_ emissions, has a high mortality rate. This association still needs to be better investigated in the North Region, especially due to the high rates of deforestation, which can increase the incidence of malaria by modifying the habitats of vector mosquitoes. Another point for discussion concerns the inherent limitation of our study design, where “CO_2_ levels” may be a confounding variable and “living in a rural area” (with low CO_2_ levels) is the true associated variable. Therefore, we emphasize the importance of Brazilian information systems to granularize the level of information to better tailor public policies to the rural population.

Regarding deforestation, this study revealed high mortality rates from the disease in the North Region. According to the study of Laporta et al. [33], deforestation in previously densely forested areas is related to the increase in larval habitat due to human occupation in settlements, where contact with the host occurs, which leads to the migration of the vector to the newly occupied areas. This may explain the high mortality rates found in the North, highlighting the need for integrated public policies that combine environmental preservation, socioeconomic development, and the strengthening of the health system in the most affected areas.

Regarding urban sanitation, the IBGE sanitation distribution reports show a lack of sanitation availability in the North and Northeast Regions. Although the South region has low rates of sewage from conventional collection networks, it has different urban sanitation dynamics [34]. These regional characteristics corroborate the results of our study, which revealed that limited access to urban sanitation increases mortality rates in the North and Northeast regions. In contrast, greater access to urban sanitation reduces mortality rates in the South. This suggests that the data show a regional inequality in access to basic sanitation in Brazil, which may directly impact malaria mortality.

The data from this study on solid waste collection in the North and Northeast Regions show that insufficient coverage of these services is associated with a higher mortality rate. Unlike other regions of the country, where the coverage of waste collection is broad, 20% of the waste generated in the North and Northeast Regions does not have an adequate final destination. This can impair health promotion by creating environments conducive to the spread of vectors, such as those of malaria [35].

Income inequality and lower GDP per capita were associated with higher malaria mortality in the North, Northeast, and Midwest Regions. In the North, a lower GDP was a predictor of higher mortality, while the highest Gini index was observed in the North and Midwest. A lower GDP per capita may be associated with low access to health services and adequate infrastructure, increasing the population’s vulnerability to infectious diseases such as malaria [36]. In addition, greater income inequality, measured by the Gini index, is associated with worse health indicators due to the relationship between income concentration and geographical space, which limits access to essential health services by the less favored and underserved population. Data from the Ministry of Health confirm that mortality is greater in less economically developed regions [37]. These regional disparities contribute to vulnerability to malaria and high mortality rates in these areas. It is important to emphasize that these variables may be more associated with the lower availability of health professionals and consultations in economically less developed regions and may act as confounding variables.

In addition to socioeconomic disparities, the lower availability of health professionals and consultations in the North, Northeast, and Midwest Regions increases the mortality rate from malaria. This may be associated with the low primary care coverage, as noted by the Institute for Health Policy Studies, which revealed that 72.69 million people (34% of the population) do not have access to the Family Health Strategy, and 33.3 million depend exclusively on the SUS. Although the majority of this population is in capital cities, there are also vulnerable municipalities with low primary care coverage [38]. Therefore, there is a need to invest and maintain local teams to ensure effectiveness in eliminating malaria, maintain surveillance and prevent outbreaks of transmission, and train health professionals and services in the correct diagnosis of this disease [4].

In addition, lower health expenditures were observed in the Northeast and Midwest Regions, which face higher mortality rates from malaria, suggesting that inadequate infrastructure and ineffective distribution of resources aggravate the vulnerability of the population. The number of malaria cases in Brazil is associated with the lack of financial resources available for some Brazilian municipalities. According to Barreto et al. [39], areas that present high risks of malaria transmission have a low human development index (HDI) and low per capita income, which leads to lower investment in health services in these regions. In this sense, lower expenditures on malaria prevention may be associated with a greater risk of severe disease [40]. In contrast, the Southeast Region has lower mortality rates even with reduced expenditures due to the training of health professionals and services in the correct diagnosis of the disease [41]. Despite these findings in the literature, it is possible to observe that malaria mortality in the extra-Amazonian region is several times higher than in the Amazon. This is because health professionals in these states are not accustomed to diagnosing malaria and often confuse it with dengue [42]. In this sense, public policies for training and organizing professionals for public health interventions and services should consider social, cultural, and epidemiological aspects.

The limitations refer to the use of secondary data at the population level because an ecological bias is introduced when performing the analyses with aggregated data instead of individual data. In addition, data from information systems may suffer regional variations, especially regarding notifications and inaccuracies of cases, impacting the reliability of mortality estimates due to the disease. In addition, the characteristics of ecological studies make direct inferences of causality impossible, making it essential to conduct case–control or cohort studies. Even in the design of the ecological population study, different databases were used, with different temporal patterns for analysis, and annual moving averages were used to overcome the bias of spurious correlations. Other limitations that serve as guidelines for future research as well as recommendations for data quality are that more than half of the data with laboratory information on the vector were not available, which certainly implies improving access to this information in public health surveillance systems. The Supplementary Materials include scripts for data mining and data quality analysis and a spreadsheet for standardizing mortality by age in the regions.

## 5. Conclusions

This study revealed a reduction in malaria mortality in recent years, with the highest rate occurring in the 0–4 age group in the period of 2000–2004, and when the birth cohort was analyzed, the rate was greater for people born before 1960, mostly men, Indigenous people, and residents of the North Region. Reduced urbanization, low CO_2_ emissions, and limited access to urban sanitation were associated with higher mortality rates in the North, Northeast, and South Regions. Predictive models indicated that lower CO_2_ emissions are linked to higher mortality rates in the North, while greater access to urban sanitation reduces mortality rates in the South. The North and Northeast Regions, which are characterized by greater deforestation and less solid waste collection, exhibited greater mortality. Areas with lower GDP per capita and higher Gini indices in the North, Northeast, and Midwest Regions showed higher mortality, with lower GDP being a predictor in the North Region and a higher Gini index in the North and Midwest Regions. Lower availability of health professionals and consultations (North, Northeast, and Midwest), together with lower health spending (Northeast and Midwest), increased mortality in these regions, while in the Southeast Region, low spending was associated with lower mortality rates.

We therefore conclude that malaria mortality has specific characteristics related to the different regions of Brazil. Thus, this research can contribute to better targeting public policies.

## Figures and Tables

**Table 1:** Study variable classification.

Variable	Description	Primary Source	Secondary Source	Collection Period
Dependent				
Age-standardized mortality rate.	Obtained using the crude mortality rate in the population, adjusted according to the age group of the study’s standard population to indicate the mortality that a population would have if it presented a standard age structure, allowing accurate comparisons of malaria mortality.	DATASUS	SIM	1996–2022
Independent				
Regions	Malaria deaths according to region of residence, based on the country’s regional divisions (North, Northeast, South, Southeast, and Midwest).	DATASUS	SIM	1996–2022
Race/Color	Classification of the number of deaths in the studied population according to the race/color criterion (Brown, White, Black, Indigenous, and Yellow).	DATASUS	SIM	1996–2022
Urbanization	Proportion of residents living in urban areas according to the surveyed year.	DATASUS	IDB	2000–2012
Gross Domestic Product	Calculation of the sum of goods and services per person by the total population residing in the region.	DATASUS	IDB	2000–2010
Gini Index	A measure of the inequality of income distribution in a population.	DATASUS	IDB	2000–2012
Number of health professionals/inhabitant	The number of professionals living in a given region per 1000 inhabitants in the year under study.	DATASUS	IDB	2000–2010
Spending on public health actions and services	Percentage of GDP spent on health measures and services by government sector in the year under review.	DATASUS	IDB	2000–2010
Consultations/inhabitant per region	Total SUS medical visits per person in a given area and year of survey.	DATASUS	IDB	2000–2012
Anthropogenic carbon dioxide emissions	Measure of CO_2_ released into the atmosphere by human activity by sector.	IBGE	IDS	2000–2014
Number of hotspots	Many hotspots detected by satellite capturing and processing images taken in the North, Northeast, South, Southeast, and Midwest regions. in a region North, Northeast, South, Southeast, and Midwest.	IBGE	IDS	2000–2016
Annual gross deforestation in the legal Amazon/km²	Satellite-based annual estimates of deforestation rates.	IBGE	IDS	2000–2015
Percentage of garbage collection in urban areas	Percentage of households with regular garbage collection in urban areas.	IBGE	IDS	2004–2015
Percentage of garbage collection in rural areas	Percentage of households with regular refuse collection in rural areas.	IBGE	IDS	2004–2015
Access to sewage via a collection network is essential in urban areas.	Percentage of households regularly served by sewerage system.	IBGE	IDS	2004–2015

Mortality Information System (SIM); Basic Indicators and Data (IDB); Brazilian Institute of Geography and Statistics (IBGE); sustainable development indicators (IDS); Department of Information and Informatics of the Unified Health System (DATASUS).

**Table 2:** Descriptive analysis of the entire cohort.

	Brazil n = 1631 (%)	North n = 1159 (%)	Northeast n = 183 (%)	Southeast n = 115 (%)	South n = 39 (%)	Midwest n = 128 (%)
**Sex**						
Male	1009	682	130	74	28	95
Female	601	490	49	39	11	32
**Race**						
White	404	224	33	67	32	48
Black	112	65	18	13	3	13
Yellow	14	10	0	2	1	1
Brown	891	700	112	24	3	52
Indigenous	109	102	2	0	0	5
**Age (years)**						
0 to 4	216	204	4	1	0	7
5 to 9	61	57	3	0	0	1
10 to 14	71	55	9	0	0	7
15 to 19	108	52	18	2	1	5
20 to 29	232	175	22	10	8	17
30 to 39	212	135	34	19	8	16
40 to 49	211	124	23	26	10	28
50 to 59	198	119	22	28	8	21
60 to 69	118	81	11	11	2	13
≥70	197	62	16	7	0	8
Unknown	7	7	0	0	0	0
**Marital status**						
Single	701	522	77	26	19	57
Married	417	241	68	54	12	42
Widowed	94	67	10	10	0	7
Separated	33	8	0	14	4	7
Other	56	47	6	1	0	95

**Table 3:** Spearman correlation between the sustainable development indicators and mortality rate.

Dimensions	North	Northeast	Southeast	South	Midwest
*Environmental*					
Urbanization	−0.845 *	−0.854 *	−0.022	−0.747 *	−0.394
Anthropogenic carbon dioxide emissions	−0.776 *	−0.857 *	−0.402	−0.914 *	−0.474
Number of hotspots	0.447	0.113	−0.544	−0.267	0.408
Annual gross deforestation in the legal Amazon/km²	0.702 *	0.873 *	0.198	0.539	0.331
Percentage of garbage collection in urban areas	−0.785 *	−0.769 *	0.411	−0.405	−0.280
Percentage of garbage collection in rural areas	−0.365	−0.790 *	0.191	−0.586	0.016
Access to public sewage	−0.870 *	−0.787 *	−0.283	−0.747 *	0.529
*Socioeconomics*					
GDP	−0.709 *	−0.634 *	0.408	0.088	−0.574 *
Gini Index	0.578 *	0.649 *	−0.419	0.012	0.842 *
*Institutional*					
Number of health professionals/inhabitant	−0.688 *	−0.657 *	0.416	0.057	−0.712 *
Spending on public health actions and services	−0.285	−0.706 *	0.625 *	−0.140	−0.709 *
Consultations/inhabitant per region	−0.713 *	−0.566 *	0.456	0.013	−0.631 *

GDP—Gross Domestic Product. * *p* < 0.05.

**Table 4:** Linear regression of sustainable development indicators predictors of mortality.

Coefficients Model	Standardized Coefficients Beta	T	*p*
***Environmental dimension***			
*North*			
(Constant)		4.506	0.004
Anthropogenic carbon dioxide emissions	−0.879	−5.866	0.001
Number of hotspots	−0.121	−0.783	0.463
Percentage of garbage collection in rural areas	0.183	1.161	0.29
*South*			
(Constant)		−1.949	0.087
Access to public sewage	−1.401	−3.676	0.006
Percentage of garbage collection in rural areas	0.76	1.995	0.081
***Socioeconomic dimension***			
*North*			
(Constant)		−0.835	0.428
GDP	−0.6223	−3.203	0.013
Gini Index	0.461	2.37	0.045
*Midwest*			
(Constant)		−3.105	0.015
GDP	−0.236	−1.195	0.266
Gini Index	0.734	3.711	0.006

GDP—Gross Domestic Product.

## Data Availability

All data were made available at https://opendatasus.saude.gov.br/dataset (accessed on 12 June 2024).

## Supplementary Materials

The supporting information can be downloaded at: https://www.mdpi.com/article/10.3390/tropicalmed10020041/s1.
